# Insufficient mycophenolate mofetil maintenance dosage increases the relapse risk in neuromyelitis optica spectrum disorders

**DOI:** 10.3389/fimmu.2026.1815301

**Published:** 2026-05-21

**Authors:** Xingyue Zhou, Jing Du, Yinghui Duan, Chenyi Shi, Jinjing Jia, Hanqing Gao, Xinyi Xiao, Shugang Cao, Qun Xue

**Affiliations:** 1 Department of Neurology, First Affiliated Hospital of Soochow University, Suzhou, China; 2 Department of Neurology, Second Affiliated Hospital of Anhui Medical University, Hefei, China; 3 Clinical Research Center of Neurology, Jiangsu Institute of Clinical Immunology, The First Affiliated Hospital of Soochow University, Suzhou, China

**Keywords:** neuromyelitis optica spectrum disorders, mycophenolate mofetil, corticosteroids, relapse, dose-effect

## Abstract

**Objective:**

The utilization of biologic agents for neuromyelitis optica spectrum disorders (NMOSD) is constrained by high cost and limited accessibility in China. While clinical guidelines recommend mycophenolate mofetil (MMF) for relapse prevention, the factors influencing its therapeutic efficacy remain poorly characterized. This study aimed to investigate clinical predictors of relapse in patients with NMOSD treated with MMF, specifically evaluating the impact of MMF maintenance dosage and concurrent corticosteroid therapy on relapse prevention.

**Methods:**

In this retrospective cohort study, clinical data were collected from NMOSD patients consecutively treated with MMF at the Departments of Neurology of the First Affiliated Hospital of Soochow University and the Second Affiliated Hospital of Anhui Medical University. Patients were categorized into three groups based on their maintenance dosage: low-dose (≤ 750 mg/d), medium-dose (= 1000 mg/d), and high-dose (≥ 1250 mg/d). The primary endpoint was the first relapse during treatment. Kaplan-Meier analysis was employed to compare relapse-free survival rates among groups, and Cox proportional hazards regression models were utilized to identify factors associated with relapse during follow-up.

**Results:**

A total of 82 NMOSD patients were included (34 males and 48 females), with a mean age of 43.6 ± 15.9 years. During the median follow-up time of 27.7 months, 39 patients (47.6%) experienced relapse. Kaplan-Meier analysis demonstrated that the relapse-free survival rate in the high-dose group was significantly higher than that in the medium-dose group and the low-dose group (log-rank test, *p* = 0.003). Additionally, concurrent corticosteroid use was linked to a reduced risk of relapse (*p* = 0.008, log-rank test). Multivariate Cox proportional hazards regression analysis indicated that compared with the high-dose group, patients in the medium-dose group (HR = 4.724, 95% CI: 1.537-14.522, *p* = 0.007) and low-dose group (HR = 4.758, 95% CI: 1.631-13.878, *p* = 0.004), as well as those who had previously received other conventional immunosuppressants (HR = 2.057, 95% CI: 1.038-4.073, *p* = 0.039), had a significantly increased risk of relapse. Conversely, concurrent corticosteroid use (HR = 0.417, 95% CI: 0.207-0.840, *p* = 0.014) emerged as a protective factor against relapse.

**Conclusion:**

Suboptimal MMF maintenance dosing (≤ 1000 mg/d) significantly increases the risk of relapse in patients with NMOSD. A high-dose regimen (≥ 1250 mg/d) and concurrent corticosteroid administration, if well-tolerated, represent key strategies to mitigate relapse risk during MMF treatment.

## Introduction

Neuromyelitis optica spectrum disorders (NMOSD) constitute a group of autoimmune inflammatory demyelinating diseases of the central nervous system (CNS), characterized by high relapse rates and severe disability. Clinical manifestations include optic neuritis (ON), acute myelitis (AM), area postrema syndrome (APS), brainstem syndromes (BS), cerebral syndromes (CS), and diencephalic syndromes (DS). Approximately 70% - 80% of NMOSD patients test positive for aquaporin-4 antibody (AQP4-Ab) (1). Epidemiological data indicate a higher prevalence of NMOSD in Asian populations, with a predilection for young and middle-aged women (2). As the disease duration extends and the number of relapses increases, NMOSD patients often experience a stepwise accumulation of disability (3).

The primary therapeutic objectives in NMOSD are the mitigation of acute symptoms and the early initiation of immunotherapy to prevent relapses. Although the 2025 Chinese guidelines for the diagnosis and treatment of NMOSD recommend biologic agents—including B-cell depleting agents, IL-6 receptor blockers, and complement inhibitors—as first-line therapies, conventional immunosuppressants remain a clinical mainstay due to the high cost and administration barriers associated with biologics (4). Among these, mycophenolate mofetil (MMF), a non-steroidal immunosuppressant, exerts anti-inflammatory effects and reduces relapse risk by selectively inhibiting T- and B-lymphocyte proliferation. While current domestic and international guidelines suggest an MMF maintenance dose of 1000–2000 mg/d (4–6), a definitive consensus on the optimal maintenance dosage and tapering strategy remains elusive. Previous retrospective studies have demonstrated that MMF effectively reduces the annual relapse rate in NMOSD, maintaining efficacy and safety while facilitating corticosteroid-sparing (7). However, whether different MMF dosing regimens result in disparate outcomes in relapse prevention remains inconclusive. Clinical observations suggest that patients on low-dose maintenance or monotherapy (following corticosteroid withdrawal) may face an elevated risk of relapse (8–10); furthermore, substantial dosage reduction after achieving clinical stability may also trigger disease activity. We hypothesized that MMF dosage and its combination with corticosteroids are critically linked to the risk of NMOSD relapse. Beyond the pharmacologic regimen, clinical factors such as age at onset, phenotypic manifestations, history of prior attacks, and AQP4-Ab status are implicated in disease relapse and disability progression, although reported findings remain inconsistent (11–13). Consequently, this study investigated the factors associated with relapse in NMOSD patients treated with MMF, aiming to optimize its clinical application and inform personalized treatment strategies.

## Patients and methods

### Study population

This retrospective cohort study collected data from NMOSD patients treated with MMF during the remission phase at the outpatient and inpatient departments of Neurology in the First Affiliated Hospital of Soochow University and the Second Affiliated Hospital of Anhui Medical University between January 1, 2017 and January 31, 2026. The study protocol was approved by the Institutional Review Boards of both participating centers, and written informed consent was obtained from all participants or their legal guardians. Inclusion criteria were: (1) fulfillment of the 2015 International Panel for NMO Diagnosis (IPND) criteria (5); (2) availability of comprehensive clinical records, including longitudinal medical histories, serological/imaging data, detailed medication records, and documented relapse events; and (3) maintenance of a stable MMF regimen during remission for at least three months. Exclusion criteria included: (1) presence of severe systemic comorbidities (e.g., hepatic/renal failure, cardiovascular disease, or malignancies) or psychiatric disorders; (2) suboptimal initial MMF dosing (starting dose <1000 mg/d); (3) inadequate follow-up (< 6 months); (4) prior exposure to biologic agents preceding MMF initiation; or (5) incomplete clinical documentation.

### Methods

#### Clinical and laboratory data

Comprehensive clinical profiles were abstracted for all patients. Demographic baselines (gender, onset age) were paired with data on initial clinical manifestations (ON, AM, APS, BS, CS, or DS). We also screened for autoimmune comorbidities, including systemic lupus erythematosus (SLE), Sjögren’s syndrome, and other rheumatological or thyroid disorders. Regarding therapeutic management, we reconstructed the treatment history to capture both acute interventions (e.g., intravenous methylprednisolone, intravenous immunoglobulin, plasma exchange, or immunoadsorption) and long-term maintenance strategies. Special emphasis was placed on the pre-MMF phase, specifically documenting disease duration, relapse burden (attacks in the prior 1–2 years), and baseline annualized relapse rate (ARR) before MMF initiation. Additionally, the history of previous immunosuppressant use and the status of concomitant corticosteroid tapering at the study endpoint were recorded. Diagnostic data included CNS imaging (brain/spine/orbital MRI) and laboratory panels covering AQP4-Ab serostatus, ANA/ANCA profiles, antiphospholipid antibody, and thyroid function.

#### MMF treatment regimen

All enrolled patients initiated MMF treatment during the remission phase or when corticosteroids were switched to oral administration. MMF was administered orally twice daily on an empty stomach. The baseline maintenance dose was established at a minimum of 1000 mg/d, with upward titration to 1250–1500 mg/d permitted based on individual tolerance and disease severity. Initially, all patients received combined corticosteroid therapy with a minimum maintenance dose of 2.5–10 mg per day. To mitigate risks such as myelosuppression and hepatotoxicity, a tiered safety monitoring protocol was implemented. Complete blood counts and liver function tests were assessed biweekly during the first month, monthly thereafter, and every 3–6 months during long-term follow-up if no adverse events occurred.

For patients demonstrating sustained clinical stability for more than 1–2 years, the protocol allowed for gradual corticosteroid withdrawal or prudent MMF tapering (maintaining ≥1000 mg/d). However, strictly adhering to this protocol proved challenging in real-world settings; a subset of patients unilaterally reduced dosages or discontinued corticosteroids due to safety concerns or side effects. Consequently, to reflect real-world usage, patients were stratified based on previous studies (4–6, 9) and their actual regimen at the final follow-up: the low-dose group (≤ 750 mg/d), medium-dose group (= 1000 mg/d), and high-dose group (≥ 1250 mg/d).

#### Outcome assessment

Patients were followed from the initiation of MMF therapy through January 31, 2026, with the first post-treatment relapse serving as the primary endpoint. Consistent with established protocols (14), a relapse event was adjudicated based on the acute onset of new neurological deficits or the exacerbation of pre-existing symptoms persisting beyond 24 hours. Crucially, these events were required to occur at least 30 days after the preceding attack and be distinct from pseudo-relapses triggered by infection, hyperthermia, or other systemic disturbances. Upon confirmation of a relapse, we documented the relapse date, clinical manifestations, corresponding MRI evidence, and the concurrent MMF or corticosteroid dosage. Patients who died, were lost to follow-up, or switched to an alternative immunotherapy were right-censored at the time of the respective event or their last clinical assessment. The time-to-event (or censoring) was calculated from the initiation of MMF therapy to the first documented clinical relapse or the date of censoring.

### Statistical analysis

Statistical analysis was performed using SPSS 22.0 software. The Kolmogorov-Smirnov test was used for normality testing. Normally distributed quantitative data were expressed as mean ± standard deviation and compared using the independent samples t-test. Non-normally distributed quantitative data were expressed as median (M) and interquartile range (IQR) and compared using the Mann-Whitney U test or the Kruskal-Wallis H test. Categorical variables were reported as frequencies (percentages) and compared using the Chi-square test or Fisher’s exact test, as appropriate. To identify independent predictors of relapse, we constructed Cox proportional hazards regression models that incorporated variables with p-values approaching significance (*p* < 0.1) in univariate analysis. A significance threshold of *p* < 0.1 was employed as the screening criterion for multivariable analysis to prevent the premature exclusion of potentially relevant clinical confounders, an established approach in exploratory modeling. Multicollinearity among covariates was assessed using Variance Inflation Factors (VIFs) prior to their inclusion in the multivariable Cox proportional hazards model, with a VIF < 5 confirming the absence of significant multicollinearity. Hazard ratios (HR) and 95% confidence intervals (CI) were calculated. A two-sided p < 0.05 was considered statistically significant. Data visualization was performed using GraphPad Prism (version 8.0) or Microsoft PowerPoint.

## Results

### Baseline characteristics

**Figure 1:**
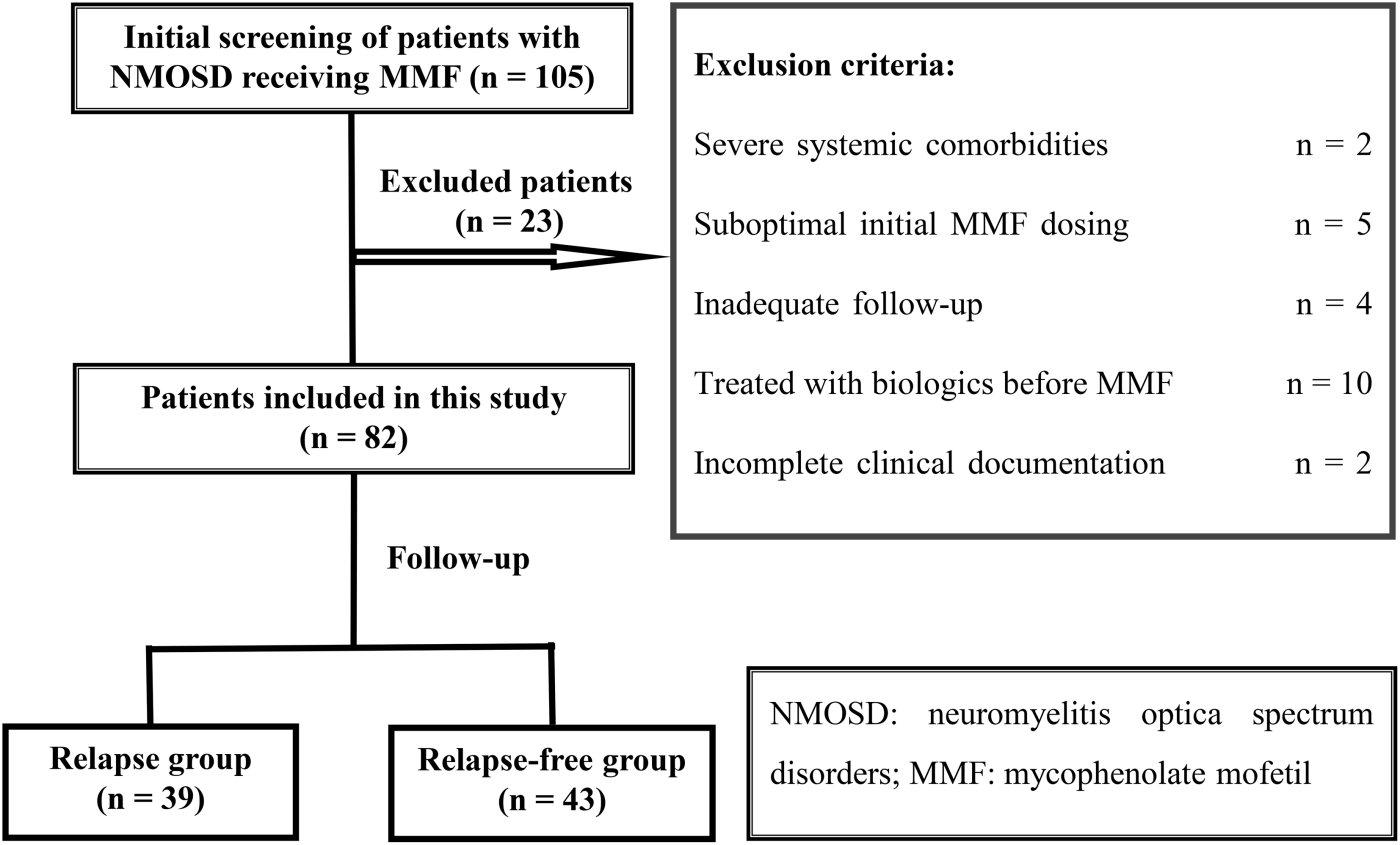
Study flow diagram.

Of the 105 patients with NMOSD initially screened, 82 satisfied the eligibility criteria and were included in the final analysis (34 males and 48 females; mean [SD] age, 43.6 [15.9] years; range, 16–79 years) (**Figure 1**). MMF initiation occurred after a median disease duration of 26.3 months (IQR: 13.4, 50.8). Among the cohort, 75 patients (91.5%) were seropositive for AQP4-IgG. Concurrent autoimmune disorders were present in 17 patients, including Sjögren’s syndrome (n = 6), SLE (n = 3), undifferentiated connective tissue disease (n = 2), and isolated cases of vasculitis, rheumatoid arthritis, antiphospholipid syndrome, systemic sclerosis, Sjögren’s syndrome with autoimmune thyroiditis, and Sjögren’s syndrome with SLE. The predominant initial clinical manifestations were AM (45.1%) and ON (41.5%), followed by less frequent BS (3.7%), APS (2.4%), and CS (2.4%), as well as multiple syndromes (4.9%). Patients were stratified by final MMF maintenance dosage into three groups: low-dose (n = 18), medium-dose (n = 33), and high-dose (n = 31). Over a median follow-up of 27.7 months (IQR: 14.3, 53.5), 39 patients (47.6%) experienced a relapse. Notably, the relapse rate was significantly lower in the high-dose group (12.9%, 4/31) compared with the medium (66.7%, 22/33) and low-dose groups (72.2%, 13/18).

### Comparison of baseline characteristics among MMF dosage groups

Baseline characteristics were generally comparable across the three dosage groups. No statistically significant differences were identified regarding age at onset, sex distribution, AQP4-Ab seropositivity, concurrent autoimmune disorders, or the prevalence of autoantibody positivity. Similarly, clinical manifestations, disease duration prior to MMF initiation, total pre-treatment attacks, and attack frequency prior to MMF were similar among groups (all *p* > 0.05). However, a significant difference was observed in optic nerve involvement prior to MMF initiation (*p* = 0.021), with a lower prevalence in the high-dose group (32.3%) compared with the low- (55.6%) and medium-dose groups (66.7%) (**Table 1**).

**Table 1:** Differences between the MMF dose groups.

Variables	Low-dose (n =18)	Medium-dose (n = 33)	High-dose (n = 31)	*P*-value
Onset age, years	44.8±15.2	42.4±15.7	44.2±15.4	0.846
Gender (female), n (%)	11 (61.1)	21 (63.6)	16 (51.6)	0.602
AQP4-Ab positivity, n (%)	17 (94.4)	31 (93.9)	27 (87.1)	0.543
Autoimmune comorbidities, n (%)	6 (33.3)	8 (24.2)	3 (9.7)	0.117
Autoimmune antibody seropositivity, n (%)	8 (44.4)	11 (33.3)	11 (35.5)	0.724
Number of autoantibody positives	0 (0, 3)	0 (0, 1)	0 (0, 1)	0.517
Initial clinical manifestations, n (%)				0.253
ON	10 (55.6)	17 (51.5)	7 (22.6)	
AM	6 (33.3)	13 (39.4)	18 (58.1)	
APS/BS/CS/DS	1 (5.6)	2 (6.1)	4 (12.9)	
Multiple symptoms	1 (5.6)	1 (3.0)	2 (6.5)	
Clinical manifestations before MMF, n (%)				0.133
ON	4 (22.2)	8 (24.2)	1 (3.2)	
AM	6 (33.3)	9 (27.3)	15 (48.4)	
APS/BS/CS/DS	0 (0)	1 (3.0)	3 (9.7)	
Multiple symptoms	8 (44.4)	15 (45.5)	12 (38.7)	
Multisite involvement, n (%)	8 (44.4)	15 (45.5)	12 (38.7)	0.849
Spinal cord involvement, n (%)	13 (72.2)	23 (69.7)	27 (87.1)	0.224
Optic nerve involvement, n (%)	10 (55.6)	22 (66.7)	10 (32.3)	0.021
Brain involvement, n (%)	4 (22.2)	7 (21.2)	7 (22.6)	0.991
Relapse within one year before MMF, n (%)	11 (61.1)	19 (57.6)	16 (51.6)	0.792
Disease duration before MMF, months	20.4 (6.7, 52.3)	9.3 (0.9, 59.6)	12.2 (3.0, 53.2)	0.406
Total number of attacks before MMF	2 (1, 4)	2 (1, 3)	2 (1, 3)	0.676
Number of attacks in the prior 2 years before MMF	1 (1, 1)	1 (1, 1)	1 (1, 1)	0.875
Number of attacks in the prior 1 years before MMF	1 (1, 1)	1 (1, 1)	1 (1, 1)	0.915
ARR before MMF initiation	0.5 (0, 0.7)	0.3 (0, 1.0)	0.3 (0, 1.0)	0.727
Prior use of other immunosuppressants, n (%)	6 (33.3)	9 (27.3)	5 (16.1)	0.354
MMF initiated upon relapse, n (%)	11 (61.1)	17 (51.5)	18 (58.1)	0.773
Concurrent use of corticosteroids, n (%)	15 (83.3)	24 (72.7)	28 (90.3)	0.187
Relapse, n (%)	13 (72.2)	22 (66.7)	4 (12.9)	<0.001

AQP4-Ab, aquaporin-4 antibodies; MMF, mycophenolate mofetil; ON, optic neuritis; AM, acute myelitis; APS, area postrema syndromes; BS, brainstem syndromes; CS, cerebral syndromes; DS, diencephalic syndromes; ARR, annualized relapse rate.

### Differences between the relapse and relapse-free groups

Comparative analysis between the relapse (n = 39) and relapse-free (n = 43) groups revealed statistically significant differences in AQP4-Ab positivity (*p* = 0.025), concurrent use of corticosteroids (*p* = 0.005), and MMF maintenance dose (*p* < 0.001). Baseline demographics and disease characteristics—including age, gender, autoimmune comorbidities, disease duration, and pre-MMF disease activity (ARR and prior attack frequency)—did not differ significantly between the two groups (all *p* > 0.05) (**Table 2**). Among relapsed patients, 12 switched to other immunosuppressants (6 to inebilizumab, 2 to satralizumab, 4 to rituximab), while 27 patients were maintained on MMF.

**Table 2:** Differences between the relapse and relapse-free groups.

Variables	All patients (n = 82)	Relapse-free group (n = 43)	Relapse group (n = 39)	*P*-value
Onset age, years	43.6±15.9	43.6±14.0	43.6±17.0	0.985
Gender (female), n (%)	48 (58.5)	26 (60.5)	22 (56.4)	0.710
AQP4-Ab positivity, n (%)	75 (91.5)	36 (83.7)	39 (100)	0.025
Autoimmune comorbidities, n (%)	17 (20.7)	7 (16.3)	10 (25.6)	0.296
Autoimmune antibody seropositivity, n (%)	30 (36.6)	15 (34.9)	15 (38.5)	0.737
Number of autoantibody positives	0 (0, 2)	0 (0, 1)	0 (0, 2)	0.651
Initial clinical manifestations, n (%)				0.489
ON	34 (41.5)	17 (39.5)	17 (43.6)	
AM	37 (45.1)	20 (46.5)	17 (43.6)	
APS/BS/CS/DS	7 (8.5)	5 (11.6)	2 (5.1)	
Multiple symptoms	4 (4.9)	1 (2.3)	3 (7.7)	
Clinical manifestations before MMF, n (%)				0.495
ON	13 (15.9)	6(14.0)	7 (17.9)	
AM	30 (36.6)	18(41.9)	12 (30.8)	
APS/BS/CS/DS	4 (4.9)	3 (7.0)	1 (2.6)	
Multiple symptoms	35 (42.7)	16 (37.2)	19 (48.7)	
Multisite involvement, n (%)	35 (42.7)	16 (37.2)	19 (48.7)	0.293
Spinal cord involvement, n (%)	63 (76.8)	33 (76.7)	30 (76.9)	0.985
Optic nerve involvement, n (%)	42 (51.2)	19 (44.2)	23 (59.0)	0.181
Brain involvement, n (%)	18 (22.0)	8 (18.6)	10 (25.6)	0.442
Relapse within one year before MMF, n (%)	46 (56.1)	26 (60.5)	20 (51.3)	0.403
Disease duration before MMF, months	14.1 (2.4, 50.4)	12.1 (2.9, 49.5)	16.4 (1.4, 53.7)	0.985
Total number of attacks before MMF	2 (1, 3)	2 (1, 3)	2 (1, 3)	0.969
Number of attacks in the prior 2 years before MMF	1 (1, 2)	1 (1, 2)	1 (1, 2)	0.895
Number of attacks in the prior 1 years before MMF	1 (1, 1)	1 (1, 1)	1 (1, 1)	0.366
ARR before MMF initiation	0.3 (0, 1)	0.3 (0, 1)	0.4 (0, 0.9)	0.799
Prior use of other immunosuppressants, n (%)	20 (24.4)	7 (16.3)	13 (33.3)	0.073
MMF initiated upon relapse, n (%)	49 (59.8)	27 (62.8)	22 (56.4)	0.556
Concurrent use of corticosteroids, n (%)	67 (81.7)	40 (93.0)	27 (69.2)	0.005
MMF maintenance dosage, n (%)				<0.001
High-dose	31 (37.8)	27 (62.8)	4 (10.3)	
Medium-dose	33 (40.2)	11 (25.6)	22 (56.4)	
Low-dose	18 (22.0)	5 (11.6)	13 (33.3)	

AQP4-Ab, aquaporin-4 antibodies; MMF, mycophenolate mofetil; ON, optic neuritis; AM, acute myelitis; APS, area postrema syndromes; BS, brainstem syndromes; CS, cerebral syndromes; DS, diencephalic syndromes; ARR, annualized relapse rate.

### Analysis of risk factors for relapse during MMF treatment

**Figure 2:**
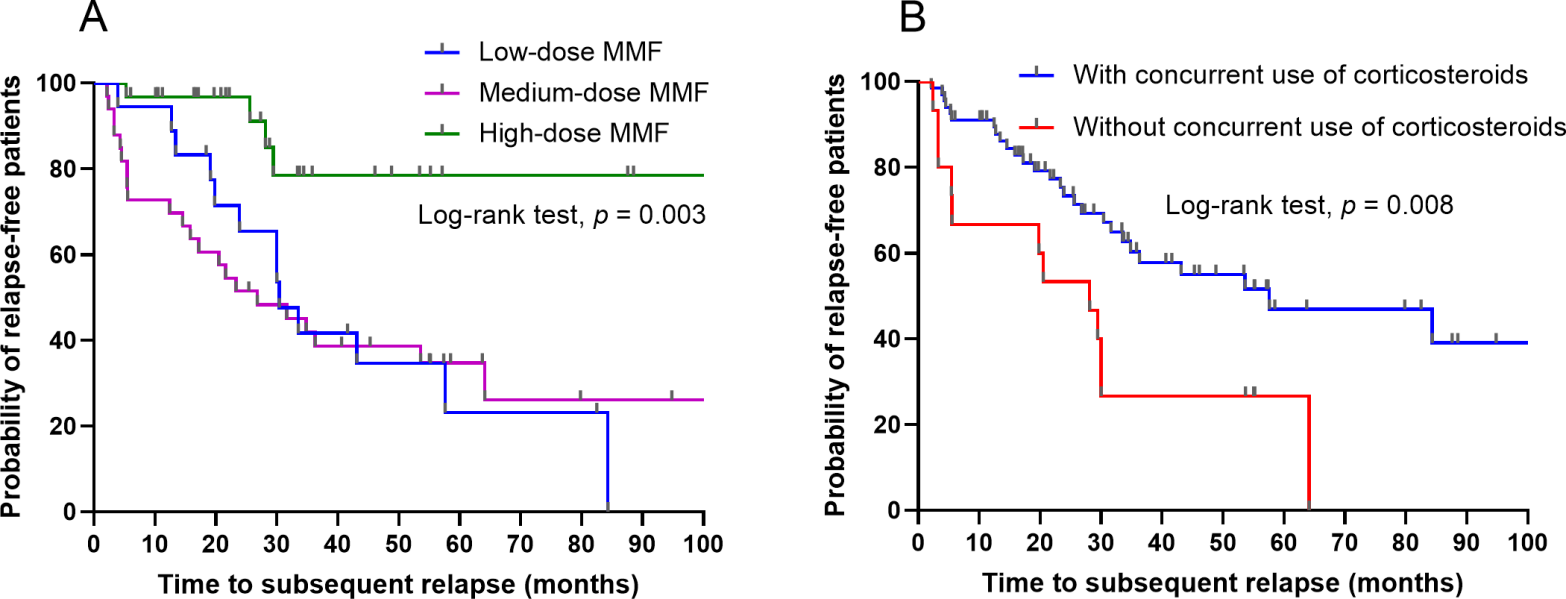
Kaplan-Meier estimates of relapse-free survival. **(A)** Comparison across different mycophenolate mofetil (MMF) dosage groups. **(B)** Comparison between patients with and without concurrent use of corticosteroids.

Kaplan-Meier analysis demonstrated statistically significant differences in relapse-free survival rate among the three MMF dose groups (*p* = 0.003, log-rank test). Specifically, patients in the medium- and low-dose groups exhibited a significantly higher risk of relapse compared with the high-dose group (**Figure 2A**). Additionally, the concurrent use of corticosteroids was linked to a reduced risk of relapse (*p* = 0.008, log-rank test). Specifically, patients treated with MMF in combination with corticosteroids exhibited a significantly higher probability of relapse-free survival compared to those receiving MMF alone (**Figure 2B**).

In the univariate Cox proportional hazards regression, the concurrent use of corticosteroids (HR = 0.408, 95% CI: 0.205-0.810, *p* = 0.010), medium MMF dose (HR = 4.939, 95% CI: 1.608-15.172, *p* = 0.005), low MMF dose (HR = 5.187, 95% CI: 1.784-15.083, *p* = 0.003), and prior use of other immunosuppressants (HR = 1.935, 95% CI: 0.991-3.777, *p* = 0.053) were identified as potential predictors (threshold *p* < 0.1). These variables were subsequently entered into the multivariate Cox regression model. Multivariate analysis identified the concurrent use of corticosteroids (HR = 0.417, 95% CI: 0.207–0.840, *p* = 0.014) as an independent protective factor against relapse. Conversely, prior use of other immunosuppressants (HR = 2.057, 95% CI: 1.038–4.073, *p* = 0.039), medium MMF dosage (HR = 4.724, 95% CI: 1.537–14.522, *p* = 0.007), and low MMF dosage (HR = 4.758, 95% CI: 1.631–13.878, *p* = 0.004) were confirmed as independent risk factors for relapse (**Table 3**).

**Table 3:** Analysis of risk factors for relapse during MMF treatment.

Variables	Univariate analysis		Multivariate analysis	
	HR (95% CI)	*p*-value	HR (95% CI)	*p*-value
Onset age	1.006 (0.984-1.029)	0.603	–	–
Gender	1.636 (0.847-3.158)	0.143	–	–
AQP4-IgG positivity	23.848 (0.232-2455.291)	0.180	–	–
Autoimmune comorbidities	1.177 (0.572-2.421)	0.658	–	–
Autoimmune antibody seropositivity	1.114 (0.584-2.125)	0.744	–	–
Number of autoantibody positives	1.037 (0.846-1.270)	0.729	–	–
Initial clinical manifestations			–	–
ON	Ref			
AM	1.558 (0.778-3.119)	0.211		
APS/BS/CS/DS	0.616 (0.142-2.670)	0.518		
Multiple symptoms	2.758(0.786-9.677)	0.113		
Clinical manifestations before MMF			–	–
ON	Ref			
AM	1.344 (0.519-3.477)	0.542		
APS/BS/CS/DS	0.522 (0.064-4.249)	0.544		
Multiple symptoms	1.379 (0.576-3.303)	0.471		
Multisite involvement	1.229 (0.655-2.305)	0.520	–	–
Spinal cord involvement	1.438 (0.675-3.063)	0.346	–	–
Optic nerve involvement	1.076 (0.564-2.051)	0.824	–	–
Brain involvement	1.315 (0.638-2.707)	0.458	–	–
Relapse within one year before MMF	0.726 (0.385-1.370)	0.323	–	–
Disease duration before MMF	1.000 (0.993-1.007)	0.980	–	–
Total number of attacks before MMF	0.985 (0.876-1.107)	0.796	–	–
Number of attacks in the prior 2 years before MMF	0.832 (0.433-1.598)	0.834	–	–
Number of attacks in the prior 1 years before MMF	0.854 (0.455-1.604)	0.581	–	–
ARR before MMF initiation	0.917 (0.603-1.394)	0.684	–	–
Prior use of other immunosuppressants	1.935 (0.991-3.777)	0.053	2.057 (1.038-4.073)	0.039
MMF initiated upon relapse	0.786 (0.415-1.488)	0.460	–	–
Concurrent use of corticosteroids	0.408 (0.205-0.810)	0.010	0.417 (0.207-0.840)	0.014
MMF maintenance dosage				
High-dose	Ref			
Medium-dose	4.939 (1.608-15.172)	0.005	4.724 (1.537-14.522)	0.007
Low-dose	5.187 (1.784-15.083)	0.003	4.758 (1.631-13.878)	0.004

AQP4-Ab, aquaporin-4 antibodies; MMF, mycophenolate mofetil; ON, optic neuritis; AM, acute myelitis; APS, area postrema syndromes; BS, brainstem syndromes; CS, cerebral syndromes; DS, diencephalic syndromes; ARR, annualized relapse rate.

### Sensitivity analysis

To assess the robustness of our findings and mitigate potential confounding during the MMF induction phase, we performed a sensitivity analysis in which patients who experienced a relapse within the first 6 months of MMF treatment were censored at the 6-month mark. Following this adjustment, Kaplan-Meier analysis revealed that the relapse-free survival rate remained significantly higher in the high-dose group compared with the medium- and low-dose groups (log-rank *p* = 0.012). Furthermore, multivariable Cox proportional hazards regression confirmed that, relative to the high-dose group, the medium-dose (HR = 3.870, 95% CI: 1.099–13.622, *p* = 0.035) and low-dose (HR = 5.713, 95% CI: 1.608–20.292, *p* = 0.007) groups maintained a significantly elevated risk of relapse. These results strongly align with the primary analysis, thereby substantiating the robustness of our conclusions.

## Discussion

Since NMOSD is a CNS inflammatory disorder characterized by a high ARR and significant disability accumulation, the cornerstone of long-term management is the effective prevention of relapses (15, 16). Although MMF is a conventional immunosuppressant widely used for this condition, the optimal maintenance strategy remains poorly defined. In this retrospective study, we demonstrated that compared with a high-dose maintenance regimen (≥ 1250 mg/d), the use of medium or low doses served as an independent risk factor for relapse. Conversely, the concurrent use of corticosteroids was identified as a protective factor. These findings provide critical clinical evidence for optimizing MMF treatment strategies in NMOSD.

Although current guidelines recommend an MMF dosage of 1000–2000 mg/d, typically overlapped with corticosteroids for the first 3 months, the optimal maintenance dose remains a subject of debate (4–6). Retrospective evidence from Jiao et al. identified a dose-dependent efficacy, showing that moderate doses (1250–1500 mg/d) achieved significantly lower relapse rates than lower regimens (≤1000 mg/d; 48% vs. 91%, *p* = 0.031), thereby proposing ≥1250 mg/d as a therapeutic threshold (9). Conversely, Huang et al. suggested that a low-dose regimen (1000 mg/d) could effectively maintain remission when combined with low-dose corticosteroids (17). This ambiguity is further reflected in a recent meta-analysis, which confirmed MMF’s overall efficacy in reducing the ARR but noted substantial heterogeneity regarding the effective dose range (1000–2000 mg/d) (7). Given the lack of consensus regarding optimal MMF maintenance dosing and the imperative to balance efficacy with safety, identifying an evidence-based dosing strategy is of paramount clinical importance. In the present study, guided by existing literature (4–6, 9) and the prevalence of real-world underdosing, we stratified patients into three dose cohorts. Our findings demonstrate a definitive dose-response relationship, wherein patients receiving low (≤ 750 mg/d) or medium (1000 mg/d) maintenance doses carried a significantly increased risk of relapse compared with those in the high-dose group (≥ 1250 mg/d). This reinforces the ≥1250 mg/d threshold as essential for sustained clinical stability (9). These divergent findings likely stem from variations in patient demographics and neuroimmunological phenotypes, alongside non-standardized dose stratification across studies. Furthermore, the clinical context of corticosteroid administration—specifically the proportion of patients on dual therapy and the specific maintenance dosages used—plays a pivotal role. Such pharmacological synergy could potentially account for the maintained remission observed at lower MMF dosages in certain cohorts, a factor that must be rigorously accounted for in cross-study evaluations.

Mealy et al. reported a 36.3% failure rate for MMF, which was lower than that of azathioprine but significantly higher than rituximab (18). Furthermore, recent data from Bilodeau et al. (2026) confirmed that patients treated with MMF or azathioprine face a substantially higher risk of relapse (HR: 1.75 and 2.33, respectively) compared with rituximab-treated cohorts (19). While we acknowledge that rituximab or other recently approved disease-modifying therapies (DMTs) should be prioritized when accessible, our study focuses on optimizing MMF therapy in clinical settings where these first-line options remain constrained. Our data provide clinical justification for revising current dosing paradigms; specifically, we propose that a maintenance threshold of at least 1250 mg/d is necessary to mitigate the risk of breakthrough relapse secondary to suboptimal immunosuppression.

Additionally, our study underscores the benefit of using corticosteroids concurrently. While minimizing long-term steroid exposure and achieving steroid-free remission remain primary therapeutic goals (20, 21), our data demonstrate that the concurrent use of corticosteroids yields superior efficacy compared with MMF monotherapy. This clinical advantage may be attributed to the complementary mechanisms of corticosteroids, which induce lymphocyte apoptosis and exert broad anti-inflammatory effects, whereas MMF selectively inhibits lymphocyte proliferation (22, 23). Consequently, for high-risk patients (e.g., those with a high pre-treatment ARR) or those unable to tolerate optimal MMF dosages, we recommend extending the duration of concomitant low-dose corticosteroid therapy or adopting a more gradual tapering regimen. However, detailed corticosteroid tapering schedules were not documented, precluding the evaluation of potential dose–duration synergy with MMF. Beyond the current maintenance strategy, a patient’s prior therapeutic exposure also emerged as a significant predictor of relapse risk. Specifically, the administration of non-corticosteroid immunosuppressants (e.g., azathioprine, cyclophosphamide) prior to MMF initiation was identified as a risk factor for relapse. This subgroup likely comprises patients with refractory or aggressive disease phenotypes who exhibited suboptimal responses to previous treatments. Consistent with the findings of San Martin et al. (13) and Bilodeau et al. (19), prior immunosuppressant exposure serves as a clinical marker for patients with intrinsically refractory disease who remain at an elevated relapse risk even after transitioning to alternative therapies. Their immunopathological mechanisms might be more complex or active, leading to a higher relapse risk even after switching to MMF. Consequently, patients switching to MMF following therapeutic failure may warrant intensified maintenance regimens—such as biologic agents or high-dose MMF combined with corticosteroids—alongside vigilant clinical monitoring.

This study has several limitations that should be acknowledged. First, the retrospective design and small sample size preclude comprehensive control over unmeasured confounders (e.g., medication adherence, infection triggers). Second, the empirical MMF dose stratification, although informed by existing literature (9), warrants validation in larger prospective trials. Third, real-world clinical variability (e.g., discrepancies in MMF initiation timing and corticosteroid regimens) may introduce residual confounding. Fourth, lacking data on mycophenolic acid concentrations and lymphocyte subsets precludes fully elucidating underlying pharmacokinetic and pharmacodynamic mechanisms. Fifth, unsystematic adverse event documentation prevents comparative tolerability evaluation; nevertheless, high-dose MMF (1750–2000 mg/d) exhibits a recognized safety profile in NMOSD (7, 9), and consequently, the relatively lower doses used in our study generally yielded no reports of severe adverse events. Finally, fixed-dose stratification unadjusted for body weight or individual pharmacokinetics may influence systemic drug exposure and limit generalizability. Despite these caveats, our results’ concordance with prior studies (7, 9, 17) and robust sensitivity analyses substantiate our conclusions. Nonetheless, findings should be cautiously extrapolated to populations with differing disease durations or activities.

## Conclusion

Suboptimal MMF maintenance dosing (≤ 1000 mg/d) significantly increases the risk of relapse in patients with NMOSD. A high-dose regimen (≥ 1250 mg/d) and concurrent corticosteroid administration, if well-tolerated, represent key strategies to mitigate relapse risk during MMF treatment. Future research should also focus on therapeutic drug monitoring to delineate the precise therapeutic window and the optimal duration of corticosteroid combination.

## Data Availability

The raw data supporting the conclusions of this article will be made available by the authors, without undue reservation.
